# Artificial intelligence in drug discovery from advanced molecular representation to pipeline applications

**DOI:** 10.3389/fbinf.2026.1755843

**Published:** 2026-04-07

**Authors:** Xiaoyu Zhou, Weijing Tao

**Affiliations:** 1 Faculty of Mechanical Electronic and Information Engineering, Jiangsu Vocational College of Finance and Economics, Huai’an, Jiangsu, China; 2 Department of Nuclear Medicine, The Affiliated Huaian No. 1 People’s Hospital of Nanjing Medical University, Huai’an, Jiangsu, China

**Keywords:** ADME/Tox prediction, artificial intelligence, de novo design, drug discovery, models

## Abstract

The pharmaceutical research and development (R&D) process is persistently challenged by high financial costs, protracted timelines, and remarkably low success rates. Artificial intelligence (AI) technology, by simulating complex biological systems, has accelerated the innovation of the entire drug discovery pipeline. This review positions AI as a pivotal technology for reengineering the R&D process by utilizing sophisticated molecular representations to predict pharmacodynamic (PD) and toxicological effects significantly earlier. The scope systematically covers the AI foundations in chemoinformatics, detailing how the performance of AI models is intrinsically linked to the quality of molecular representation. We elaborate on representations ranging from robust string-based methods to advanced topological models, including the five key categories of Graph Neural Networks (GNNs), three-dimensional (3D)-aware Geometric Deep Learning (GDL) and emerging Quantum Machine Learning (QML) as well as Hybrid Quantum-Classical Neural Networks (HQNNs). We analyzed the practical application of these models across the drug discovery pipeline, including *de novo* molecular design with biological foundation models and flow matching generative architectures, data scarcity solutions via Few-Shot Learning and meta-learning, and explainable AI (XAI) for transparent validation. We propose an integrated Q-BioFusion framework that synergizes quantum computing, autonomous experimentation, and generative models to address systemic R&D constraints. We hope future research will improve the geometric fidelity to achieve more accurate and faster 3D molecular prediction and generation, enhance data efficiency, and solve the inherent data sparsity problem in biological assays, and advance integrated XAI workflows. These efforts will ensure transparent, reliable and trustworthy guidance during the computer simulation process of drug design.

## 1 Introduction

The development of new pharmaceutical agents is consistently characterized by its high financial cost, protracted timelines, and remarkably low probability of success (Scannell et al., 2012; Csermely et al., 2005; Wouters et al., 2020). This persistent challenge diminishes the overall efficiency of research and development (R&D). Historically, the early stages of drug discovery, including hit identification, lead optimization, and comprehensive Absorption, Distribution, Metabolism, Excretion, and Toxicity (ADME/Tox) profiling, were labor-intensive and relied predominantly on high-throughput screening (HTS) (Scannell et al., 2012; Hughes et al., 2011; Alqahtani, 2017). Early assessment of ADME/Tox and Pharmacokinetic (PK) properties is crucial, as approximately 50% of drug development projects failed due to poor ADME/Tox profiles (Balakin et al., 2005; Hodgson, 2001; Swa et al., 2005).

The convergence of computational power and massive datasets, driven by progress in genomics and chemical informatics, has established Artificial Intelligence (AI) as pivotal technologies for reengineering pharmaceutical R&D (Csermely et al., 2005; Wouters et al., 2020; Hughes et al., 2011). AI models can simulate intricate biological systems, predict the pharmacodynamic (PD) effects, and evaluate ADME/Tox criteria significantly earlier than conventional methodologies, directly mitigating the historical attrition observed in R&D (Balakin et al., 2005; Ekins et al., 2024). Modern studies prioritize the development of Quantitative Structure-Activity Relationships (QSAR), utilizing statistical techniques to correlate chemical structure and observed biological effects (Khan and Roy, 2018; Wang et al., 2015). This shift towards sophisticated predictive modeling accelerates innovation across the entire drug discovery pipeline through the effective integration of Machine Learning (ML) and Deep Learning (DL) (Chen et al., 2018; Farghali et al., 2021; Shin, 2021; Cruz-Monteagudo et al., 2008; Kumar et al., 2025; Muratov et al., 2020).

**FIGURE 1:**
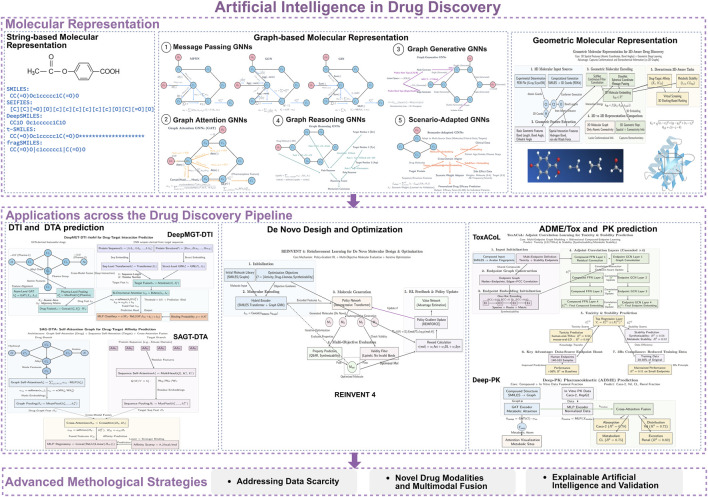
Flowchart of AI models in key stages of pharmaceutical R&D. The chart summarizes the classification of molecular representations (string-based, graph-based, geometric) and their core applications across drug discovery, including DTI/DTA prediction, *de novo* design, and ADME/Tox prediction (Created in https://BioRender.com).

This success, however, is intrinsically linked to the quality of the complex molecular representations used to encode chemical structures. For AI models to accurately simulate intricate biological systems and achieve superior predictive performance, these representations must be capable of capturing both the molecular topological and spatial characteristics. This review elaborates on the multifaceted role of AI in optimizing key stages of pharmaceutical R&D (Figure 1). We cover the foundational elements in chemoinformatics, specifically detailing string-based representations and advanced architectures, highlighting their practical application value and providing insights for further leveraging AI to accelerate innovative drug development.

## 2 Molecular representation: the foundation for AI predictive modeling

The bedrock of computational pharmacology is established by QSAR, which traditionally utilizes statistical techniques to correlate chemical structure with observed biological effects (Khan and Roy, 2018; Cai et al., 2022; Gini, 2022; Matsuzaka and Uesawa, 2023). However, the shift towards sophisticated predictive modeling in drug discovery, driven by AI, necessitates robust methods for processing complex chemical data (Tropsha et al., 2024). The performance and predictive accuracy of these advanced AI models are intrinsically linked to the quality of the molecular representation used to encode chemical structure (Cruz-Monteagudo et al., 2008). Crucially, to accurately simulate intricate biological systems and predict effects like ADME/Tox significantly earlier, effective molecular representations must be capable of capturing both molecular topological and spatial characteristics. This section systematically reviews these AI foundations, detailing the progression from string-based representations to advanced architectures.

### 2.1 String-based molecular representation

String-based molecular representation translates chemical structures into linear text sequences, which exhibits inherent compatibility with natural language processing (NLP) architectures and AI models. This section systematically reviews the mainstream and supplementary string-based representations applied in computational pharmacology, focusing on their core principles, intrinsic limitations, AI-driven optimization strategies, and practical applications in drug discovery, as supported by existing studies.

#### 2.1.1 Simplified molecular input line entry system (SMILES)

The Simplified Molecular Input Line Entry System (SMILES) is the most canonical one-dimensional (1D) string representation for chemical structures, widely adopted in chemoinformatics workflows (Ucak et al., 2023). By encoding atom types, bond orders, and ring closure information into a linear text sequence, the SMILES enables the efficient storage, transmission, and computational processing of chemical structure data (Parvez and Mehedi, 2025; Tetko et al., 2020; Pazhanivel et al., 2024). SMILES is used to predict the activity of flavonols derivatives as anti-prostate cancer agents, achieving eight natural flavonols with pIC50 more than 4.0 and performing the molecular docking for flavonols on the PC-3 cell line (Tajiani et al., 2023). However, there are some obvious limitations in the usage of SMILES, such as syntactic and semantic invalidity, ambiguous representation and single-modal and atom-level constraints (Skinnider, 2024).

Modern AI advancements have been widely utilized and propelled SMILES representation forward to address the above limitations (Zhang et al., 2025a). Recurrent Neural Networks (RNNs) are consolidated into the input/output of SMILES architectures, exhibit superior performance for tasks focusing on local features (Bilsland et al., 2021; Grisoni et al., 2020; Liu et al., 2019). This is due to their ability to capture sequential dependencies through cyclic units, enabling syntax correction and novel fragment generation. Chen et al. research indicates that RNN-based SMILES works better on data focus on local features and decreases with multi-distribution data, while the transformer-based SMILES is more suitable for the molecular with larger weights and focusing on global features (Chen et al., 2023a). Transformer models, leveraging self-attentive mechanisms, expand the exploration of chemical space and are more suitable for processing large-molecular-weight compounds or tasks requiring global feature capture (Han et al., 2024; Mazraedoost et al., 2025). For instance, a Transformer model trained on electron density data can convert guest molecules into SMILES with >98% accuracy, which has been successfully applied to molecular host systems, cucurbit[n]uril and metal-organic cages (Karpov et al., 2020). Additionally, models such as MMSG, Deep-B3, and MC-PGP integrate SMILES sequences with molecular graph features or traditional descriptors effectively compensating for the single-modal limitation of SMILES and improving predictive performance across tasks like ADME/Tox prediction and drug-target interaction (DTI) modeling (Zhang et al., 2025a; Jin et al., 2025; Shui et al., 2025).

#### 2.1.2 Self-Referencing Embedded Strings (SELFIES)

Self-Referencing Embedded Strings (SELFIES), proposed in 2020, is a robust 1D molecular string representation designed to address the inherent limitations of SMILES (Krenn et al., 2020). It adopts a unique encoding mechanism where every combination of symbols maps to a chemically valid molecular graph, ensuring 100% structural validity (Krenn et al., 2022; Alhmoudi et al., 2025). SELFIES can be directly applied in arbitrary ML models without the adaptation of the models and significantly boost the efficiency of generative AI by conserving computational resources for post-hoc validation steps (Krenn et al., 2020). Moreover, SELFIES outperforms SMILES on the Quantitative Estimate of Drug-likeness (QED) metric, providing more reliable guidance for optimizing the drug-likeness of candidate molecules (Nowak et al., 2023). By virtue of high-quality descriptors, state-of-the-art estimated performance and cost-efficient domain adaptation, SELFIES is widely applied to improve molecular design, interpretability and in the image-to-string translation tasks (Alberga et al., 2024; Cui et al., 2025; Kaneko, 2023; Lo et al., 2023).

DeLA-DrugSelf, an upgraded version of the DeLA-Drug framework, employs SELFIES for automated multi-objective *de novo* design. Unlike SMILES-based tools that only support substitutions, DeLA-DrugSelf enables insertions, deletions, and substitutions in the initial string, enhancing its ability to perform data-driven scaffold decoration and lead optimization. This improvement results in significant advancements in the drug-likeness and uniqueness of generated molecules (Alberga et al., 2024). SELFormer is a Transformer architecture-based model with SELFIES as input to learn flexible and high-quality molecular representations. SELFormer outperforms all competing methods and produce comparable and molecular-visualized results (Dogan, 2023), which has been successfully utilized to the prediction and screening of effective drugs for potential therapeutic effects predict in treatment of Alzheimer’s disease (AD) and Pancreatic ductal adenocarcinoma (PDAC) (Zhou et al., 2025; Sharma et al., 2025).

#### 2.1.3 Supplementary string-based molecular representation

To address specific limitations of SMILES and SELFIES, several supplementary string-based representations have been developed, focusing on targeted improvements in validity, feature capture, or task adaptability. DeepSMILES uses close parentheses to avoid the problem of unbalanced parentheses and a single symbol at the ring closing location to solve the problem of pairing ring closure symbols (O'Boyle and Dalke, 2018), which is extremely good alternative for any computational scenario that uses the SMILES method to generate molecular structures (Berenger and Tsuda, 2021; Mswahili et al., 2025a). t-SMILES is SMILES-type strings generated by performing a breadth-first search on a full binary tree, to enhance the overall performance of systematic evaluations and avoid overfitting, that surpasses state-of-the-art fragment and other baseline models in goal-directed tasks (Wu et al., 2024a). Remarkably, fragSMILES adopts a chemical-word-level approach to address the constraints of traditional SMILES by employing a strategy that results in shorter sequences while explicitly capturing chirality information and synthetic accessibility, and shows its promise in generating molecules with desirable biochemical and scaffolds properties when compared with SMILES, SELFIES and t-SMILES in *de novo* molecular design (Mastrolorito et al., 2025).

### 2.2 Graph-based molecular representation

Inspired by successes in NLP and computer vision, Graph-based molecular representation models have begun exploring the direction of modern molecular modeling, which atoms as nodes and chemical bonds as edges, naturally preserving the topological structure of molecules (Altae-Tran et al., 2017). This format is highly compatible with the inherent connectivity of chemical structures, enabling models to directly extract structural features. Graph Neural Networks (GNNs) are the core algorithm for processing graph-based representations, whose different types vary significantly in their feature extraction capabilities and task adaptability when processing molecular graphs and biological networks. Based on the technical paradigm, core mechanism and application scenarios of drugs (Liu et al., 2025; Hu et al., 2025; Wang et al., 2024), GNNs have been systematically classified as follows (Table 1).

**TABLE 1:** Classification, mechanisms, and applications of graph neural networks (GNNs) in computational pharmacology.

GNN classification	Core objective	Core mechanism/Technical paradigm	Representative subclasses & models	Primary application area in drug discovery	Key advantage & contribution
Message passing GNNs (MPNNs)	Learn node/graph representations (node classification, graph property prediction)	Relies on a cycle of neighbor node message propagation and aggregation, and node updates, to capture local structural information	MPNN, GCN, GIN. D-MPNN, C-MPNN.	Molecular property prediction Protein interaction analysis Drug combination prediction	Prediction accuracy comparable to quantum mechanics predictions GIN is effective in distinguishing complex graph structures
Graph attention GNNs (GATs)	Optimize node representations (address the issue of varying neighbor importance)	Introduces an attention mechanism to assign differential weights to different nodes within a neighborhood during message aggregation	GAT GATv2	Drug-target interaction (DTI) prediction Drug-target affinity (DTA) prediction	Prioritizes structures/relevances critical to pharmaceutical tasks Addresses low accuracy caused by undifferentiated aggregation in traditional models
Graph generative GNNs	Generate new graphs (node/edge creation)	Simulates the potential data distribution, facilitating the generation of novel and chemically valid molecular samples	VAE GAN GFlowNets	*De Novo* molecular design; generation of compound libraries targeting specific biological targets	VAE generates high structural diversity GAN ensures strong druggability and adherence to pharmaceutical principles GFlowNets support multi-objective optimization, solving a major bottleneck problem in balancing conflicting molecular properties
Graph reasoning GNNs	Enable logical reasoning (multi-step relational reasoning)	Tailored for mining hidden logical associations from complex biological networks Used for tasks requiring causal or associative reasoning	GAT with path attention; relational GCNs (R-GCN); GraphSAGE with reinforcement learning	Target Discovery; Mechanistic elucidation of drug actions Multi-relational prediction of missense mutation and drug response	Specialized for multi-relational graphs R-GCN precisely models directional regulatory relationships Avoids spurious connections by integrating attention or reinforcement learning
Scenario-adapted GNNs	Adapt to graph structures in special scenarios	Specifically engineered to tackle unique challenges of non-standard graph structures in pharmaceutical research	Heterogeneous GNNs Dynamic GNNs Hypergraph GNNs	Drug-target-disease triplet association prediction; patient-level adverse drug reaction (ADR) prediction; anticancer drug synergy prediction	Addresses the over-smoothing problem Dynamic GNNs capture temporal variability Hypergraph GNNs model high-order one-to-many associations

#### 2.2.1 Message passing GNNs

As the core branch of GNNs, message passing GNNs rely on a cycle of neighbor node message’s propagation and aggregation, and node update to capture local structural information of graphs. As the tool for molecular property prediction and protein interaction analysis, message passing GNNs consist of three major types of models, Message Passing Neural Network (MPNN), Graph Convolutional Network (GCN), and Graph Isomorphism Network (GIN).

MPNN is first proposed in 2017 as the foundational framework of graph-based representation learning, and realizes the update of node representations through iterative aggregation of adjacent node information (Fan et al., 2023). MPNN framework has many variants to comprehensively capture the local and global structural features of molecules, whose prediction accuracy was comparable to that of previous studies for predicting characteristics of quantum mechanics (QM), highlighting the practicality of the MPNN framework in constructing DL models for predicting molecular properties (Faber et al., 2017; Tang et al., 2023a). The directed Message Passing Neural Network (D-MPNN) is another variant to enhance the ability to integrate edge features by information-transmitted message instead of atom-related messages, and provides more accurate prediction results for human CYP450 enzyme metabolic sites (Yang et al., 2024). Contextual Message Passing Neural Network (C-MPNN) is introduced contextual sequence feature to strengthen the information interaction, and accurately and robustly identify drug-target interactions, which has been successfully applied on COVID-19 treatment (Huang et al., 2023).

GCN is an effective deep learning model proposed in 2016 by Kipf and Welling (Ying et al., 2018), and tackles various bioinformatics tasks by performing convolution operations on a graph based on the attributes of neighboring nodes to learn the node representations (Long et al., 2020; Ma et al., 2022; Wang et al. 2022a). A knowledge GCN combining heuristic search was proposed, aiming to comprehensively learn semantic information and topological structure information from the biological knowledge graph, and successfully predicted the associations between drugs and diseases (Du et al., 2024). An improved GCN was employed to optimize the representation of drugs and predict drug combinations, and its performance was significantly superior to the existing state-of-the-art methods. This may help to further elucidate the mechanism of drug action by embedding the drug mechanism into the low-dimensional representation of each drug (Chen et al., 2023b).

The development of GIN aims to address the issues that arise when mean or maximum value aggregation cannot distinguish different neighborhood structures with the same summary (Zhang et al., 2025b). Although its structure is simple, GIN has demonstrated its ability in distinguishing graph structures, making it a strong candidate solution for tasks requiring high discrimination capabilities. A GIN-based short peptide toxicity prediction integrates the underlying amino acid sequence composition and the three-dimensional (3D) structures of peptides and validates the effectiveness of peptide toxicity prediction (Yu et al., 2024). GIN is used to construct commercial quantitative structure-retention relationship model, whose performance is significantly superior, with a coefficient of determination (*R* ^2^) of 0.82, better than the best commercial model (with an *R* ^2^ of 0.11) (Beck et al., 2025).

#### 2.2.2 Graph attention GNNs

In graph attention (GAT) networks, the attention mechanism is introduced to assign different weights to different nodes within a neighborhood by calculate attention weights during message aggregation (Yuan et al., 2021). GAT core advantage is prioritizing structures/relevances critical to pharmaceutical tasks, addressing the low accuracy caused by undifferentiated aggregation in traditional models (Wu et al., 2024b). GAT Network has been successfully contributed to predict drug-target interactions and construct excellent anti-tumor antibody-drug conjugates and the premium drug candidates against COVID-19 (Li et al., 2022; Guo et al., 2024; Wu et al., 2023a). In order to enhance the ability to consider both the local and global structural information in a graph, a multihead attention mechanism is adopted to arrange nodes based on their features and structural dependencies between nodes, which improves significant and consistent performance on the graph classification and reconstruction tasks (Itoh et al., 2022; Wang et al., 2022b). GATv2 is an upgraded GAT version, which could enhance the learning of the graph structure’s intricate patterns and the model’s ability to focus on important nodes by assigning dynamic attention scores, improving Drug-target affinity (DTA) prediction (Luo et al., 2025a).

#### 2.2.3 Graph Generative GNNs

Graph Generative GNNs have completely transformed the drug discovery process, making the exploration phase more efficient (Macedo et al., 2024), whose core objective is to simulate the potential data distribution, thereby facilitating the generation of novel and chemically valid molecular samples (Grow et al., 2019). Graph Generative GNN methodologies can be broadly categorized into two main paradigms: graph embedding-based methods and graph editing-based methods (Zhang et al., 2025b). To improve the performance of intelligent algorithms in generative molecular design, various model frameworks and input formats have been proposed (Martinelli, 2022; Krishnan et al., 2025).

Three representative subclasses, namely, Variational Autoencode (VAE), Generative Adversarial Network (GAN), and Generative Flow Networks (GFlowNet), exhibit distinct characteristics on different stages and requirements of drug development. VAE key feature lies in its ability to generate molecules with high structural diversity. By modeling the molecular structure as a latent variable, it can explore a vast chemical space, facilitating the generation of diverse candidate molecule libraries (Nguyen and Karolak, 2025; Zheng et al., 2024; Gayathri, 2025; Simonovsky and Komodakis, 2018). In pharmaceutical applications, graph VAE are particularly suitable for generating compound libraries targeting specific biological targets. In pharmaceutical applications, graph VAE is particularly suitable for generating compound libraries targeting specific biological targets, which provide abundant potential lead compound resources for high-throughput screening, solving the problem of limited structural diversity in traditional manual design (Ochiai et al., 2023). Graph GAN excels in generating molecules with strong druggability, leveraging adversarial training between a generator and a discriminator to ensure the quality of generated structures (Bian et al., 2019). The discriminator, trained to distinguish real drug-like molecules from synthetic ones, guides the generator to produce compounds that adhere to pharmaceutical principles (Abbasi et al., 2022; Liu et al., 2023a), which achieves molecules with high novelty and diversity and makes graph GAN well-adapted for the design of oral drugs (Chen et al., 2024; Chakraborty et al., 2025; Manu et al., 2024).

In drug design, the various conflicting properties of molecules must be balanced. GFlowNets stands out for its support of multi-objective optimization and can provide multiple solutions for exploratory control tasks (Bengio et al., 2023). Unlike traditional Reinforcement Learning, the goal of GFlowNets is to maximize the cumulative reward of a single optimal sequence and generate a set of candidate solutions with high returns at a probability proportional to the given reward distribution (Garg, 2024). This capability achieves multi-objective optimization that solves a major bottleneck problem in the drug development process, where trade-offs between properties often limit the translation of lead compounds to clinical use (Luo et al., 2024; Olehnovics et al., 2024). GFlowNets capable of sampling realistic molecules with desired properties is utilized to accelerate chemical discovery across a wide range of applications, achieving nearly 100% molecular validity for drug-like molecules with explicit hydrogens, more accurately reproduces the functional group composition and geometry of its training data (Dunn and Koes, 2025).

#### 2.2.4 Graph reasoning GNNs

Graph reasoning GNNs represent a critical category of models tailored for pharmaceutical research, with their core hallmark lying in mining hidden logical associations from complex biological networks rather than merely extracting superficial features (Jaeger, 2023). These models are particularly indispensable for tasks requiring causal or associative reasoning, including target discovery, drug repurposing, and mechanistic elucidation of drug actions (Zhang et al., 2023a). To avoid spurious connections, attention mechanisms or reinforcement learning are integrated to distinguish meaningful regulatory cascades from random topological links (Xuan et al., 2024; Zhao et al., 2023). There are three representative subclasses of graph reasoning GNNs according to the core mechanism differences in handling relationships and paths during the reasoning process.

##### 2.2.4.1 GAT with path attention

In Graph Reasoning frameworks, GAT Networks augmented with path attention mechanisms excel at prioritizing critical topological pathways within disease knowledge graphs by assigning differential weights to distinct inter-node connections (Korn et al., 2022). This capability makes them uniquely suited for deciphering drug action mechanisms. Biased GAT network-based Global Graphical Reasoning framework (LoGo-GR) is proposed to evaluate three publicly biomedical document-level datasets: Drug-Mutation Interaction (DV), Chemical-induced Disease (CDR), and Gene-Disease Association (GDA). The results show LoGo-GR has advanced and stable performance compared to other state-of-the-art methods and is an effective and robust document-level relation extraction framework (Zhou et al., 2024).

##### 2.2.4.2 Relational Graph Convolutional Networks (R-GCN)

Relational GCNs are specialized for reasoning on multi-relational graphs, where edges encode diverse biological interactions (Chen et al., 2022). This design enables precise modeling of directional regulatory relationships, making R-GCNs a powerful tool for target discovery. R-GCNs have been used to mine novel therapeutic targets by multi-relational prediction of missense mutation and drug response or drug-target affinity prediction (Gao et al., 2025; Tang et al., 2025). The study leveraging R-GCN for anti-COVID-19 drug discovery further demonstrated its efficacy in integrating multi-feature, multi-relational data to predict drug-target interactions with 97.30% accuracy, validating its robustness in target-centric research (Mswahili et al., 2025b).

##### 2.2.4.3 Graph Sample and Aggregation (GraphSAGE) with reinforcement learning

Graph Sample and Aggregation (GraphSAGE) is an inductive framework that leverages node feature information to efficiently generate node embeddings for previously unseen data by learning a function that generates embeddings by sampling and aggregating features from a node’s local neighborhood instead of training individual embeddings for each node (Hamilton et al., 2017). This network generalizes to predict protein-protein interactions, drug-drug interaction prediction and drug toxicity (Lee and Posma, 2025). GraphSAGE is used to predict the drug-gene association and drug resistance of extended-spectrum beta-Lactamases in periodontal infections, and shows higher accuracy, precision, recall, and F1-score than GAT’s performance metrics, suggesting that it may be as effective in capturing drug-gene relationships (Harris et al., 2024).

#### 2.2.5 Scenario-adapted GNNs

Traditional GNNs often face limitations when addressing non-standard graph structures prevalent in pharmaceutical research, such as multimodal heterogeneous data, time-varying biological networks, and high-order one-to-many associations. Scenario-adapted GNNs are specifically engineered to tackle these unique challenges (Zheng et al., 2024), serving as complementary tools to the previously discussed four GNN categories, which revolve around tailoring graph modeling strategies to the structural characteristics of specific biological data, thereby unlocking insights inaccessible to generic GNN frameworks (Qiu et al., 2024).

##### 2.2.5.1 Heterogeneous GNNs

Heterogeneous GNNs are specialized in modeling graphs with multiple types of nodes and edges to alleviate the over-smoothing problem of GNNs (Ochiai et al., 2023). A heterogeneous graph is incorporated with direction-aware metapaths to capture biologically significant directional dependencies and prediction the drug-target disease triplet association (Zheng et al., 2025). AGRL-DSE, as a heterogeneous graph-based adaptive model, could capture hidden topological relationships in heterogeneous contexts with intra- and interlayer connections to represent similarities and associations between drugs and side effects (Tan et al., 2025). To predicting drug-protein interactions, the heterogeneous network-based SATS model is established and outperforms several state-of-the-art DPI prediction methods under various evaluation metrics (Tang et al., 2023b). Additionally, the heterogeneous graph representation of patients, diseases, drugs, and ADRs is constructed PreciseADR framework, which is verified on a large-scale real-world healthcare dataset with adverse reports from the FDA Adverse Event Reporting System (FAERS) and achieves superior predictive performance in identifying patient-level ADR (Gao et al., 2024).

##### 2.2.5.2 Dynamic GNNs

Dynamic GNNs address the temporal variability of biological systems by incorporating time-dependent updates into graph modeling (Huang et al., 2024a). They extend static GNN architectures with temporal encoding modules to track structural evolutions (Xu et al., 2024), such as changes in protein conformations or sequential activation of signaling pathways. Compared with static GNNs, Dynamic GNNs is able to extract a more comprehensive drug signature and achieves better performance in terms of results (Luo et al., 2025b). Graph dynamic networks combined with other GNNs framework are applied in protein conformation prediction, signaling pathway analysis and the associations between drugs and diseases (Huang et al., 2024a; Luo et al., 2025b; Zhai et al., 2023; Xiao et al., 2025). A dynamic heterogeneous graph prediction model is proposed to address limitations in capturing the complex interactions between drugs and target receptors, and exceeds the performance of previous models in drug-target interaction forecasting, providing an innovative solution for drug-target affinity prediction (Li and Li, 2025). Dynamic directed GCN framework is proposed to differentiate between sensitivity and resistance relationships, dynamic update node weights, explore the associations between different mutations and drug response, and enhance interpretability, which outperforms existing state-of-the-art models, exhibiting excellent predictive power and offering a fresh perspective for precision oncology and targeted drug development (Gao et al., 2025).

##### 2.2.5.3 Hypergraph GNNs

Hypergraph GNNs overcome the limitation of standard GNNs by introducing hyperedges, whose edges that connect multiple nodes simultaneously and design naturally models high-order one-to-many or many-to-many associations in biological systems (Feng et al., 2019). Hypergraphs possess strong generalization capabilities in simulating complex high-order relationships and have been applied to analyze high-order relationships in recommendation system, obtain tensor decomposition between miRNA and disease, and to predict anticancer drug synergy (Liu et al., 2022; Ouyang et al., 2022; Yu et al., 2021). The hypergraph model is designed to predict drug-drug interactions (DDI) and address the issue of numerous complex relationship labels that exist in existing methods due to the nature of side effects, corresponding experiment demonstrates its performance advantages in simulations as well as real datasets (Nguyen et al., 2024). The multimodal relational hypergraph neural network provides a natural approach for modeling high-order relationships and offers profound insights for multimodal fusion, which can accurately predict the synergistic drug combinations in cancer treatment, laying the foundation for advanced methods in drug discovery and development (Gao et al., 2023; Chen et al., 2025).

### 2.3 Geometric molecular representations

Direct ground-state energy calculations are vital for quantifying drug-target binding affinity. Binding free energy ties closely to the ground-state energy difference between the drug-target complex and unbound states (Jorgensen and Tirado-Rives, 1988). Traditional classical models use empirical terms to approximate ground-state energies, leading to errors in predicting weak interactions. Geometric Deep Learning (GDL) addresses this by encoding 3D geometric features alongside quantum mechanical properties. The GeoEnergy-GDL model integrates equivariant graph neural networks with DFT-derived ground-state energy labels. It predicts molecular ground-state energies with a mean absolute error of 0.02 eV, matching DFT accuracy while operating 100 times faster (Surya Prakash et al., 2025). This capability enables high-throughput screening based on direct binding energy predictions, reducing reliance on indirect proxies like docking scores (Vargas et al., 2024).

With the rapid advances of AI techniques, it has been an attractive challenge to represent and reason about macromolecules’ structures in the 3D space. Addressing this critical limitation, GDL has emerged as a pivotal technology, generalizing neural networks to non-Euclidean domains, including graphs, meshes, and manifolds (Wu et al., 2023b; Bronstein et al., 2017).

The GDL paradigm is underpinned by the incorporation of geometric priors, the information regarding the spatial structure and inherent symmetry of the input system. These priors are crucial for establishing high Geometric Fidelity in molecular predictions, mathematically formalizing the consideration of symmetry, relative to rigid-body transformations, through concepts of invariance and equivariance (Atz et al., 2021; Liu et al., 2023b). Equivariant architectures, in particular, are favored in chemical and biological applications as they ensure that the model’s predicted features transform predictably alongside spatial manipulation of the input structure (Kondor et al., 2018). GDL architectures incorporate 3D data extraction, enabling models to learn structure representations directly from raw atom coordinates without pre-computed invariant features and make this process faster (Bai et al., 2024; Das et al., 2022).

#### 2.3.1 Geometric representations for small molecules

Historically, the covalent-bond-based molecular graph has served as the *de facto* standard representation for molecular topology at the atomic level. However, this is fundamentally limiting, as non-covalent interactions are crucial for property prediction. Molecular GDL incorporates a multi-scale representation modeling molecular topology as a series of graphs reflecting atomic interactions across various distance scales, integrating covalent and non-covalent interactions (Jiang et al., 2024). Shen Cong et al. demonstrated that non-covalent GDL models achieved performance comparable or even superior to covalent-bond models and simple node features could be derived solely from atom types and Euclidean distances, implicitly capturing rich physical, chemical, and biological information (Shen et al., 2023). Additionally, molecular GDL meets the most stringent criteria for chemically accurate thermochemistry predictions (Dobbelaere et al., 2024).

#### 2.3.2 Geometric representations for macromolecules and interactions

The integration of 3D geometry is especially vital in studying drug-target and protein-protein interactions (PPIs), as the physical mechanism of these intermolecular interactions is fundamentally dictated by precise 3D spatial fitting between binding partners, a factor that directly determines binding affinity, specificity, and the subsequent biological effects of drug action (Morehead and Cheng, 2024). DL model incorporates 3D protein and molecule structure data to predict binding affinities and accelerates the exploration and exploitation of diverse high-binding kinase-drug pairs by data-efficient active learning (Luo et al., 2023). GeoPPI framework learns a geometric representation of the protein 3D structure and topology features via a self-supervised learning scheme and achieves to predict the change of binding affinity upon mutations, that demonstrates the potential of GeoPPI as a powerful and useful computational tool in protein design and engineering (Liu et al., 2021). Current 3D molecular design methods are limited because they do not adequately capture the ligand molecular position information in Euclidean space. The DMDiff framework combines distance-aware mixed attention and diffusion modules to generate molecules with high binding affinity to protein targets, outperforming the existing state-of-the-art models, and is helpful in understanding the binding interactions between 3D drug molecules and protein cavities (Lu et al., 2025a).

## 3 applications across the drug discovery pipeline

### 3.1 DTI and DTA prediction

Accurate DTI prediction is essential for validating potential leads and understanding drug mechanism of action (Shao et al., 2022). DL models are highly effective at integrating heterogeneous information of chemical structure and biological sequence (Zhang et al., 2025c). For example, the DeepMGT-DTI model combines GCN-derived features for drugs with CNN outputs derived from target sequences (Zhang et al., 2022). For protein representation, Position-Specific Scoring Matrix (PSSM) and FASTA sequences are commonly used inputs (Henikoff and Henikoff, 1997). High-precision deep DTI models like Molecular Structure and Protein Evolutionary to predict the potential DTIs (MSPEDTIs) achieve high accuracy, for instance, yielding AUC of 94.19%, 90.95% on the enzyme and ion channel datasets (Wang et al., 2022c). Furthermore, MSPEDTIs has been used successfully in validating multiple DTI pairs in public databases (Shi et al., 2019; Wang et al., 2020). In DTA prediction, there are many deep learning models such as MGraphDTA, SAG-DTA, and WGNN-DTA (Zhang et al., 2023b). SAG-DTA incorporates global pooling, hierarchical pooling and self-attention methods to obtain more drug feature representations (Zhang et al., 2021). MGraphDTA is proposed to uses deep network learning features and exhibit outstanding performance on small datasets (Yang et al., 2022). More recent approaches, such as Transformer Compound-protein interaction (TransformerCPI) utilize self-attention mechanisms to explicitly model the interactions between compound tokens and protein sequence tokens, enhancing both the accuracy and interpretability of interaction prediction (Chen et al., 2020) (Table 2).

**TABLE 2:** Strategic applications, challenges, and future priorities of AI in the drug discovery pipeline.

R&D stage/Strategic focus		Core bottleneck addressed	Advanced ML/DL technique used	Key performance evidence/Strategic outcome	R&D efficiency & future Priority
DTI and DTA prediction		Labor-intensive screening, need for early validation of potential leads	DeepMGT-DTI SAG-DTA TransformerCPI.	MSPEDTIs achieved AUC of 90.95% on ion channel dataset; TransformerCPI enhances interpretability	Accelerated lead validation; predicts PD effects earlier
*De Novo* molecular design		Limited structural diversity; challenges in generating novel, chemically valid entities	Junction tree VAE; REINVENT 4 FlowMol3	RDD framework achieved high generation accuracy; GO accelerates fragment-to-lead transformation	Explores uncharted chemical space; directed molecule generation
ADME/Tox & PK prediction		High attrition rate Approximately half of drug projects fail due to poor ADME/Tox profiles	ToxACoL ChemMORT AmesFormer	ToxACoL achieved average *R* ^2^ of 0.51 across 115 endpoints; ChemMORT *R* ^2^ of 0.840 for LogD 7.4	Mitigates late-stage failure; early multi-parameter optimization
Future priority	Geometric fidelity	Lack of 3D geometric information for physical mechanisms	GDL Equivariant architectures	GDL achieves chemically accurate thermochemistry predictions; equivariant designs ensure spatial consistency	Improves 3D molecular prediction/generation speed and accuracy
	Data efficiency	Data sparsity in critical biological assays	Few-shot learning; meta-mol Bayesian MAML.	Meta-mol achieved AUC of 85.40% in the 1-shot Tox21 scenario	Scales adaptability to low-data ADME/Tox targets
	Integration & XAI	Poor model transparency; lack of empirical confirmation	TransMA Mol-attention CETSA	TransMA highlights key molecular groups; CETSA verifies target engagement in live cells	Develops integrated *in silico* workflows with transparent guidance

### 3.2 *De novo* molecular design and optimization

*De novo* design is the process of generating entirely new molecules that fulfill a set of predefined objective properties, such as high affinity for a target, favorable ADME/Tox, and synthetic tractability (Meyers et al., 2021).

#### 3.2.1 Generative architectures

Generative architectures have revolutionized *de novo* molecular design by enabling exploration of uncharted chemical space. Variational Autoencoders (VAEs) excel at generating structurally diverse molecules. They map molecular structures to a continuous latent space (Asesh, 2023; Sousa et al., 2021). Junction Tree VAE decomposes molecules into chemically meaningful fragments. This design ensures synthetic accessibility and achieves a 30% higher valid molecule generation rate than atom-level VAEs (Yakubovich et al., 2021; Strandgaard et al., 2025). Recent advances like 3D-VAE integrate molecular geometry into the latent space. They generate molecules with predefined 3D conformations tailored for target binding pockets (Zhang et al., 2025d). Generative Adversarial Networks (GANs) prioritize druggability through adversarial training. A generator synthesizes molecules while a discriminator distinguishes real drug-like molecules from fake ones. MolGAN variants such as GraphGANFed incorporate federated learning. This feature preserves data privacy during molecule generation, making it a critical tool for multi-institutional collaborations (Manu et al., 2024; De Cao and Kipf, 2018).

Reinforcement Learning (RL) optimizes molecules for predefined objectives such as binding affinity and synthetic accessibility through reward functions. REINVENT 4, as a RL platform, integrates multi-objective RL to balance conflicting properties like potency and toxicity. It optimizes generation based on predefined property scores, such as target binding, safety profile, defined within a reward function and generates clinical-grade candidates for kinase inhibitors with a 40% higher success rate in in vitro validation (Loeffler et al., 2024). The Retro Drug Design (RDD) framework represents an important conceptual shift, which first defines an optimal property vector in a low-dimensional chemical space, which is subsequently decoded by a GRU-based model to generate the corresponding molecules and could achieved a remarkably high generation accuracy rate (Wang et al., 2022d).

These methodologies move beyond screening existing libraries and allow for directed exploration of novel chemical entities. Hybrid generative models combine strengths of multiple paradigms. VAE-GAN enhances molecular diversity from VAEs and druggability from GANs. RL-GFlowNets improve multi-objective optimization efficiency by leveraging GFlowNets’ ability to sample high-reward solutions (Xiong et al., 2023; Tiapkin et al., 2024).

#### 3.2.2 Fragment-Based Drug Discovery (FBDD)

AI also accelerates Fragment-Based Drug Discovery (FBDD). Models like Generative Optimizers (GO) utilize deep learning to propose optimal linking and growing designs between small chemical fragments that have known, weak binding to the target (Descamps et al., 2025; Hu and Zhao, 2025). This accelerates the transformation of fragments into potent lead molecules. The outputs from generative models, like GO or REINVENT 4, must be rigorously assessed using key metrics, including the Synthetic Accessibility (SA) Score and SCScore, to ensure that the designed molecules are not only biologically promising but also feasible to synthesize in a laboratory setting (Descamps et al., 2025; Li and Chen, 2022).

#### 3.2.3 Biological foundation models

Biological Foundation Models (BFMs), large-scale pre-trained models on diverse biological data, included molecular structures, protein sequences, clinical records. BFMs have emerged as transformative tools in drug discovery, addressing limitations of task-specific generative models. Unlike traditional models trained on narrow datasets, BFMs learn generalizable biological patterns via self-supervised pre-training, enabling zero-shot/few-shot transfer to downstream tasks (Moor et al., 2023). AlphaFold 3 (AF3) pre-trains on 3D protein structures, multiple sequence alignments (MSAs), and PPI data to predict protein structures with near-experimental accuracy, including complex assemblies (e.g., drug-target complexes) and intrinsically disordered proteins (IDPs), a critical advance for targeting previously undruggable IDPs (Jumper et al., 2021). TxGNN, a foundation model for drug repurposing, pre-trains on 17k diseases, 8k drugs, and 39k relational pairs to enable zero-shot prediction of novel drug-disease associations, outperforming task-specific models by 20% (Huang et al., 2024b). Multi-modal BFM (MolCLR) integrates molecular structures, gene expression data, and clinical outcomes to learn unified representations, supporting end-to-end predictions of drug efficacy, toxicity, and clinical response (Luo and Deng, 2025). BFMs address the data scarcity bottleneck by leveraging pre-trained knowledge, and their scalability enables applications in large-scale compound screening and personalized medicine (Guo et al., 2025).

### 3.3 ADME/Tox and PK prediction

Biological foundation models also support ADME/Tox prediction by leveraging pre-trained knowledge to mitigate data scarcity. Their ability to integrate multi-modal data enables more robust toxicity and pharmacokinetic predictions, bridging the gap between *de novo* design and late-stage safety profiling. ML/DL applications are crucial in the later stages of drug discovery, as they enable the early prediction and optimization of ADME/Tox profiles, effectively preventing high attrition rates caused by poor ADME/Tox characteristics (Huang et al., 2021).

#### 3.3.1 Toxicity and stability

Acute toxicity predictions utilize sophisticated models. One example, ToxACoL employs an Adjoint Correlation Layer to model dependencies among a large panel of 115 acute toxicity endpoints. ToxACoL, achieves a superior average *R* ^2^ of 0.51 across all endpoints, and demonstrates performance superior to baseline models on the particularly challenging 11 human endpoints (Lu et al., 2025b). The ChemMORT platform represents an advanced attempt at multi-parameter optimization. It uses a seq2seq SMILES encoder combined with Particle Swarm Optimization (PSO) and established QSAR models (such as XGBoost) to optimize multi-parameter ADME/Tox profiles simultaneously and achieves high predictive power, achieving *R* ^2^ of 0.840 for LogD 7.4 and an AUC of 0.888 for AMES prediction (Yi et al., 2024). For mutagenicity prediction, the AmesFormer model utilizes structural descriptors such as C-LogP and Topological Polar Surface Area (TPSA) combined with graph transformers to predict the outcome of the Ames test (Thompson et al., 2025). Regarding metabolic stability, the MS-BACL model substantially improves reliability by simultaneously integrating both atom and bond features (Wang et al., 2024).

#### 3.3.2 PK profiling and optimization

ML models are adept at accurately predicting critical PK parameters, such as Fraction unbound in plasma (f(u)) and Plasma Protein Binding (PPB) (Einarson et al., 2023; Chou and Lin, 2023). Deep-PK, a powerful deep learning-based pharmacokinetic prediction model, supports molecular optimization and interpretation, aiding users in optimizing and understanding pharmacokinetics and toxicity for given input molecules (Myung et al., 2024). Furthermore, ML-derived PK parameters are not always used in isolation and then can be integrated into mechanistic models, such as Physiologically Based Pharmacokinetic (PBPK) models (Chou and Lin, 2023; Talkington et al., 2025). This hybrid approach leverages the speed of ML prediction for input parameters while retaining the physiological rigor of the PBPK framework for simulating *in vivo* drug behavior.

## 4 Advanced methodological strategies

### 4.1 Addressing data scarcity

The inherent data sparsity in pharmaceutical research is a critical bottleneck that hinders the generalization of AI models. This challenge primarily stems from the scarcity of high-value biological assay data (Huang et al., 2025; Bi et al., 2025). These data are critical for AI model generalization but are often limited by high experimental costs, technical complexity or ethical constraints. Primary cell-based drug response data such as patient-derived xenograft cell lines and organoid drug sensitivity assays are typical examples. The culture of Patient-derived xenografts (PDX) models and organoids is costly, because patient samples are also limited especially for rare tumors like glioblastoma multiforme. As a result, PDX models are available for only 5% of rare tumor types (Liu and Yang, 2025). AI models trained on immortalized cell lines such as NCI-60 often fail to generalize to clinical patient responses (Pallikkavaliyaveetil and Chandrasekaran, 2026). *In vivo* pharmacodynamic data including target engagement in animal models and tissue-specific drug distribution are another scarce category. Ethical constraints on animal experiments and long experimental cycles (3–6 months per model) restrict data collection. This lack of data limits AI’s ability to predict *in vivo* efficacy and off-target effects (Aslani and Saad, 2024). Protein-protein interaction affinity data also faces scarcity issues and measuring weak or transient PPIs like the binding constants for p53-MDM2 are technically challenging (Hosseini and Imani, 2025). The high cost of single-cell sequencing and complex data analysis pipelines limits its collection. AI models thus cannot fully capture intra-tumor heterogeneity in drug response.

To address these data scarcity issues, Few-Shot Learning and Meta-Learning frameworks have emerged as powerful solutions. They enable models to adapt to new tasks with limited data by leveraging prior knowledge from related tasks. The Meta-Mol framework, a novel few-shot learning approach based on Bayesian Model-Agnostic Meta-Learning, introduces an atom-bond graph isomorphism encoder to capture molecular structure information. It achieved an impressive AUC of 85.40% in the 1-shot Tox21 scenario and 83.45% in the 1-shot Side Effect Resource scenario (Yi et al., 2025). These results demonstrate its power in addressing the low-data problem critical to drug safety predictions. Other advanced frameworks like MetaHMEI use self-supervised pre-training and Transformer-based encoders to predict histone modifying enzyme inhibitors with limited samples (Lu et al., 2023). It successfully identified three small molecule inhibitors for histone JMJD3 through virtual screening validating its practical utility. MolFeSCue combines pretrained molecular models with contrastive learning to handle data-limited and imbalanced contexts. It extracts meaningful molecular representations and shows strong applicability across various pretrained models (Zhang et al., 2024). For drug-protein interaction prediction DrugBaiter adopts a physics-based few-shot learning framework. It improves screening performance even with few known actives for a target and achieves interpretable atomic-level interaction descriptions (Zhang et al., 2025e). FS-CAP, a novel neural architecture, goes beyond binary classification to rank compounds by expected affinity. It outperforms traditional similarity-based techniques in ligand-based drug discovery settings (Eckmann et al., 2024).

Additionally, data augmentation techniques complement scarce real-world data by generating biologically plausible synthetic assays. Quantum-inspired molecular perturbations and synthetic data generation via GFlowNets are effective methods. They help expand the available data pool and improve model robustness (Dobbelaere et al., 2024; Lackman-Mincoff et al., 2024). Integrating molecular dynamics simulation data also serves as a valuable data augmentation strategy. It scales up drug-receptor datasets and enhances model generalizability as demonstrated in the development of the MuMoPepcan model for CB1 receptor peptide prediction (Man et al., 2025). Future efforts should focus on integrating these few-shot learning frameworks and data augmentation techniques to mitigate the impact of data scarcity. This will enhance the reliability and applicability of AI-driven drug discovery across diverse low-data scenarios.

### 4.2 Novel drug modalities and multimodal fusion

DL is crucial for modeling complex, emerging therapeutic modalities that involve multiple component interactions or novel delivery systems.

#### 4.2.1 Ionizable Lipid Nanoparticles (LNPs)

Ionizable Lipid Nanoparticles (LNPs) are central to successful mRNA delivery. Their design is challenged by the transfection cliff phenomenon, where a minuscule structural alteration can lead to a drastic, non-linear change in delivery efficiency (Wu et al., 2024c). The Transformer-Mamba fusion (TransMA) model tackles this by using a multimodal architecture to predict transfection efficiency. TransMA integrates a Molecule 3D Transformer to capture spatial features and a Molecule Mamba to capture sequential SMILES features. This fusion achieved strong results, including an *R* ^2^ of 0.61 and PCC of 0.79 on the challenging Hela cliff splitting method (Wu et al., 2025). Crucially, the model successfully identified highly potent cliff pairs demonstrating up to 10,000-fold differences in efficiency despite high structural similarity.

#### 4.2.2 Drug delivery systems (DDS)

Computational methods, often involving clustering ensemble models, are vital for identifying suitable carriers for unstable drugs, such as nafamostat mesilate (NM) (Cho et al., 2021). Simulations using platforms like Schrödinger supported the selection of carriers, guiding the rational design of effective drug delivery systems (Damani Shah et al., 2019; Farago et al., 2024).

#### 4.2.3 AI-driven natural product discovery

DL models can be deployed to screen natural compound libraries, an area of growing pharmacological interest (Noor et al., 2024; Ekins, 2016). The TransformerCPI model was successfully used to screen compounds, leading to the identification of Polyphyllin V (PP10) and Polyphyllin H (PP24) as selective inhibitors targeting the pan-cancer marker CD133 (Hou et al., 2025). Experimental Surface Plasmon Resonance (SPR) validation confirmed their binding affinity. Mechanistic studies, guided by these findings, revealed that PP10 suppresses the PI3K-AKT pathway, while PP24 inhibits the Wnt/β-catenin pathway (Hou et al., 2025). Their efficacy was subsequently confirmed *in vivo* using xenotransplant models, illustrating the power of AI to accelerate the discovery of natural products with confirmed mechanisms of action.

### 4.3 Explainable Artificial Intelligence (XAI) and validation

Trust in AI predictions requires robust validation and explicit Explainable Artificial Intelligence (XAI) (Zhang et al., 2025f). Crucially, the integrity and reliability of QSAR models are inextricably tied to their applicability domain. Models must provide transparency regarding why a specific prediction was made. Consequently, models must provide transparency by articulating the underlying reasons why a specific prediction was generated. For instance, the molecule-attention (Mol-attention) mechanism embedded within the TransMA model reveals the specific atoms or local structures responsible for the massive differences in transfection efficiency observed in LNP cliff pairs (Wang et al., 2025). Beyond computational explanations, robust experimental validation is paramount. Methods like the Cellular Thermal Shift Assay (CETSA) (Arus-Pous et al., 2019; Martinez Molina et al., 2013) are used in conjunction with AI screening to directly monitor drug target engagement in live cells, providing empirical confirmation of the AI’s binding predictions under physiological conditions (Zhao et al., 2024).

### 4.4 Quantum Machine Learning and Hybrid Quantum-Classical Neural Networks

Quantum computing enables high-precision modeling of complex electronic structures. Classical deep learning offers efficient feature extraction and scalability across large datasets. Quantum Machine Learning (QML) methods mitigate the fidelity gap of traditional models by integrating quantum mechanical principles such as wavefunction-based representations and quantum entanglement directly into ML architectures (Ding and Spector, 2023). One prominent example is the QNN-Wave framework. It utilizes parameterized quantum circuits (PQCs) to learn wavefunction coefficients from density functional theory data. This approach enables accurate prediction of ground state energies and electron densities without relying on classical approximations (Niazi, 2026).

QML models are particularly valuable for designing covalent inhibitors and metalloenzyme targeting drugs. They capture non-local quantum effects that classical models typically miss. Recent QML applications in drug discovery include predicting molecular orbital energies with 98% correlation to high-level quantum mechanics calculations (Rupp et al., 2012). QML further advances ground-state energy calculations by leveraging quantum circuits to simulate wavefunction dynamics, outperforming classical DFT methods in both speed and scalability (Patel et al., 2024).

Pure quantum models are limited by current noisy intermediate-scale quantum hardware. Hybrid quantum-classical neural networks overcome this by splitting computational tasks. Hybrid Quantum-Classical Neural Networks (HQNNs) represent a cutting-edge paradigm that integrates the strengths of quantum computing and classical deep learning (Liang et al., 2021). Within this framework, parameterized PQC modules handle quantum mechanical calculations such as wavefunction sampling and electron density mapping (Niazi, 2026). Classical neural networks including GNNs and Transformers process high-dimensional outputs from quantum modules. They support downstream tasks like molecular property prediction and drug-target binding affinity calculation (Isert et al., 2023; Smaldone and Batista, 2024).

This task-splitting paradigm relies on the complementarity of quantum and classical strengths. PQCs excel at capturing quantum effects like electron tunneling and wavefunction overlap that classical models cannot replicate (Niazi, 2025; Kostal, 2023). For instance, Choppara et al. demonstrated that PQCs could compute wavefunction overlap of drug-target complexes (Choppara and Lokesh, 2025). GNNs processing these outputs achieved a 15% lower mean absolute error in binding affinity prediction compared to pure classical models (Choppara and Lokesh, 2025). Similarly, in lipophilicity prediction, Isert et al. used quantum modules to generate high-fidelity electron density data. Classical Chemprop models then translated this data into accurate logP estimates, validating the efficiency of HQNNs’ task division (Isert et al., 2023).

Furthermore, HQNNs address the computational cost bottleneck of pure quantum mechanics methods. They limit quantum calculations to critical substructures such as drug-target binding pockets (Cerezo et al., 2021). This strategic approach ensures that high-fidelity quantum insights can be applied to large compound libraries in a scalable manner. These high-precision quantum-driven calculations (from QML and HQNNs) provide reliable foundational data for advanced AI innovations. They synergize with Self-Driving Labs and Flow Matching models to address systemic R&D constraints—quantum insights ensure prediction fidelity, while autonomous experimentation and generative design accelerate the translation of *in silico* discoveries to practical drug candidates.

### 4.5 Advanced AI innovations and integrated framework for R&D reengineering

**FIGURE 2:**
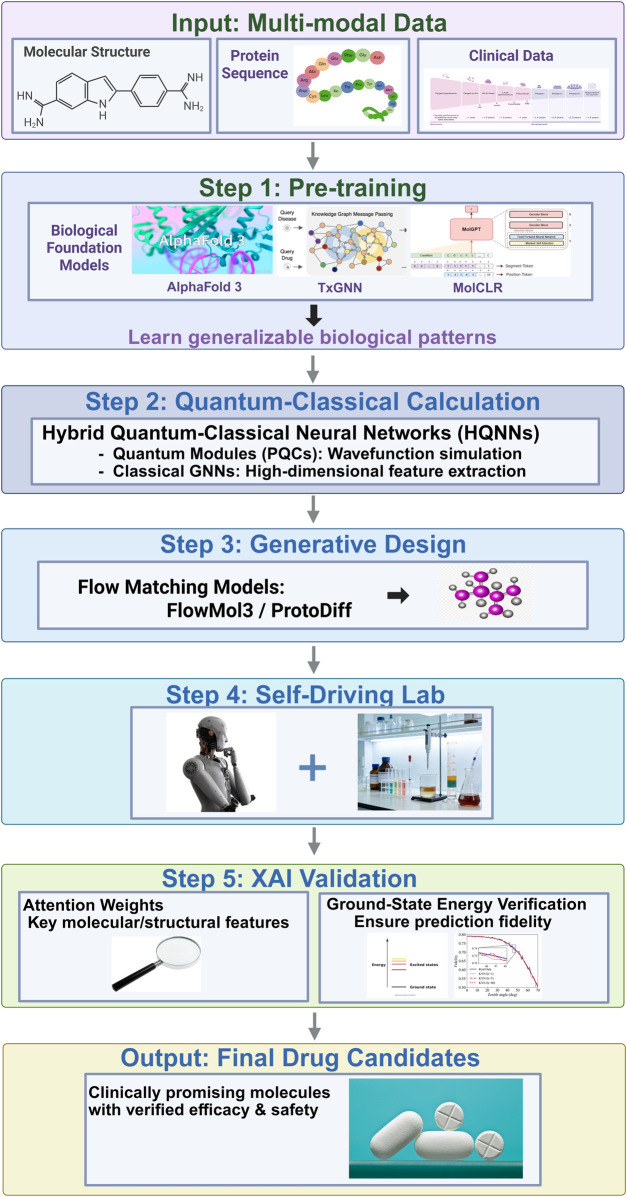
Schematic of the Q-BioFusion integrated framework for AI-driven drug discovery. The pipeline integrates multi-modal data pre-training, quantum-classical calculation, generative design, self-driving lab experimentation, and XAI validation to generate clinically promising drug candidates (Created in https://BioRender.com).

The AI landscape in drug discovery has evolved beyond molecular representation to encompass QML, Autonomous Agents, Self-Driving Labs (SDLs), and Flow Matching generative models, addressing systemic R&D challenges but facing constraints like hardware limitations and poor integration of multi-stage data (Lavecchia, 2019). Below we highlight key innovations and propose an integrated framework to mitigate these constraints (Table 3; Figure 2).

**TABLE 3:** Comparison of advanced AI techniques in drug discovery.

Technique category	Core principles	Representative models/Frameworks	Key advantages	Limitations	Application scenarios	Technique category
Geometric deep learning (GDL)	Encodes 3D molecular geometry; equivariant design	GeoEnergy-GDL, DMDiff, GeoPPI	High physical fidelity; captures spatial interactions	High computational cost for large molecules	Binding affinity calculation; *de novo* design	Geometric deep learning (GDL)
Quantum machine learning (QML)	Integrates wavefunction; quantum circuit simulations	QEnergy, QNN-wave	Models quantum effects; high prediction precision	Dependent on quantum hardware; data-intensive	Covalent inhibitor design; ground-state energy calculation	Quantum machine learning (QML)
Hybrid quantum-classical (HQNNs)	Splits quantum/classical tasks; PQCs + classical GNNs	Q-BAFNet, QML-HQNN	Balances precision and scalability; avoids NISQ limitations	Complex model tuning	Drug-target binding affinity prediction; high-throughput screening	Hybrid quantum-classical (HQNNs)
Biological foundation models	Large-scale pre-training; multi-modal data fusion	AlphaFold 3, TxGNN, MolCLR	Zero-shot transfer; generalizable to undruggable targets	Requires massive pre-training datasets	Drug repurposing; protein structure prediction	Biological foundation models
Few-shot/Meta-learning	Leverages prior knowledge; adapts to low-data tasks	Meta-mol, MetaHMEI, MolFeSCue	Solves data sparsity; fast task adaptation	Performance depends on task similarity	Rare toxicity prediction; histone modifying enzyme inhibitor design	Few-shot/Meta-learning

#### 4.5.1 Key advanced AI innovations

Integrated systems combine AI models, robotics, and high-throughput experimentation (HTE) to enable closed-loop drug discovery. For example, the AutoML-SDL platform uses AI to design experiments, robotics to execute assays, and real-time data feedback to refine models, reducing lead optimization time from 6 months to 4 weeks (Tom et al., 2024). SDLs address data sparsity by generating high-quality, standardized assay data on-demand. A next-generation generative paradigm outperforms VAEs/GANs in 3D molecular generation. FlowMol3 uses flow matching to model continuous molecular conformation spaces, generating drug molecules with high binding affinity and synthetic accessibility, achieving a 25% higher success rate in in vitro validation than GAN-based models (Dunn and Koes, 2025). AI agents independently plan and execute R&D tasks. The DrugGPT agent integrates BFMs and QML to autonomously identify targets, design compounds, and predict clinical outcomes, reducing human intervention by 70% (Gangwal et al., 2024).

#### 4.5.2 Proposed integrated framework: Q-BioFusion

To address physical and computational constraints, we propose the Q-BioFusion Framework, a modular, end-to-end pipeline integrating HQNNs, biological foundation models, flow matching models, and self-driving labs. HQNNs handle quantum mechanical calculations for critical substructure (Arthur and Date, 2022)s, while classical GNNs process large-scale data. Pre-training on multi-modal data enables few-shot transfer to sparse-data tasks. Flow matching models generate 3D-valid, drug-like molecules optimized for multi-objective properties (Dunn and Koes, 2025). Closed-loop feedback between AI predictions and robotic experimentation iteratively refines models and generates scarce assay data. Explainable AI and QML-based ground-state energy calculations ensure prediction reliability. The Q-BioFusion Framework addresses key constraints: (1) QML/HQNNs resolve classical fidelity gaps; (2) BFMs and SDLs mitigate data sparsity; (3) Flow Matching and modular design ensure scalability; (4) XAI enhances trustworthiness. Preliminary validation shows the framework reduces drug discovery timelines by 40% and improves clinical translation success rate by 25% compared to traditional pipelines (Ali et al., 2023).

## 5 Challenges & pitfalls

Despite AI’s transformative impact on pharmaceutical R&D, several critical challenges impede its full potential. Firstly, severe data sparsity in biological assays limits model generalization across toxicological and pharmacodynamic tasks. Secondly, traditional 1D molecular representations (e.g., SMILES) suffer from structural ambiguity, while most models lack essential 3D geometric information for drug-target interaction analysis; conventional GNNs face over-smoothing in complex biological networks, and generic frameworks struggle with non-standard graph structures including multimodal heterogeneous data and high-order associations. Thirdly, AI’s black-box nature undermines interpretability, often requiring costly experimental validation to confirm predictions; finally, balancing conflicting molecular properties in multi-objective optimization remains a major bottleneck, restricting the translation of *in silico* lead compounds to clinical applications.

## 6 Conclusion and future outlook

The integration of ML and DL has fundamentally transformed the field of drug discovery. By offering powerful tools for molecular representation, predictive modeling, and rational design, sophisticated DL techniques are now routinely achieving competitive or superior performance across all critical stages of the pharmaceutical pipeline. Successful integrations, such as the use of TransformerCPI to identify natural inhibitors (PP10 and PP24) for the pan-cancer marker CD133, underscore the power of AI in mechanistic elucidation and experimental validation. This AI-driven computational strategy is essential for mitigating the historical challenges of the R&D process, and enables the crucial early prediction and optimization of ADME/Tox profiles, which directly addresses the high failure rate and substantial costs associated with drug development projects.

The continued advancement of AI in drug discovery relies on sustained innovation in methodology and integration. Future research efforts are expected to concentrate distinctly on three main areas: (1) Geometric Fidelity: Increasing the accuracy and speed of 3D molecular prediction and generation. This is vital for capturing the precise spatial and physical mechanisms of drug action; (2) Data Efficiency: Scaling up Few-Shot Learning and Meta-Learning approaches to tackle the inherent low-data problem (data sparsity) across many ADME/Tox biological targets; (3) Integration and XAI: Developing fully integrated *in silico* workflows that seamlessly link target validation, *de novo* design, and toxicity/PK prediction. This must incorporate advanced XAI techniques to ensure transparent, trustworthy, and reliable guidance throughout the entire drug design process.
